# Supplementary material for: SARS CoV-2 Nucleoprotein Enhances the Infectivity of Lentiviral Spike Particles
Source: Front Cell Infect Microbiol. 2021 Apr 23;11:663688. doi: 10.3389/fcimb.2021.663688 (PMC8102828; doi:10.3389/fcimb.2021.663688)

Supplementary Information

for

SARS CoV-2 nucleoprotein enhances the infectivity of lentiviral spike particles

Tarun Mishra*[1], Sreepadmanabh M*[1], Pavitra Ramdas[1], Amit K Sahu[1], Atul Kumar[2], Ajit Chande[1,3]

Supplementary Figure 1. (A) Western blot of twenty-four SARS CoV-2 genes using an anti-strep tag antibody, with Actin serving as the loading control .(B) Normalised Luciferase readout for VSV-G pseudotyped lentiviral particles under indicated conditions, along with a Western blot of the producer cells' lysate n=4±SD (C) Normalized GFP-positive cell counts for a VSV-G pseudotyped MLV vector, produced either with or without co-transfection with the N protein n=3±SD (D) Western blot indicating the presence of spike glycoprotein in the producer cell lysates for each of the indicated combinations of spike variants and N protein, detected using anti-spike antibody, with Actin serving as the loading control. (E) D614 only-normalized infectivity levels for both wild-type (D614) and mutant (G614) spike glycoproteins, with and without the presence of N protein n=6±SD. (F) ACE2-IgFc-mediated neutralization of the G614 spike mutant-enveloped lentiviral particles, produced as per the mentioned conditions and subject to treatment with the 10ug/ml of ACE2-IgFc prior to transduction n=3±SD.

Table S1: List of Plasmids

S. No.	Plasmid Name	Purpose	Notes	Source
1.	pScalps Luciferase Zsgreen	Expression of firefly luciferase and Zoanthus green fluorescent protein	Lentiviral vector with luciferase gene under SFFV promoter and Zsgreen under Cyclophilin promoter	This study. The ~~original~~ pScalps-zsGreen vector backbone was a kind gift from Prof. Massimo Pizzato.
2.	Spike del19 codon-optimized in pcDNA3.1(-)	Expression of mammalian codon optimized SARS CoV-2 spike glycoprotein	A nineteen amino acid deletion at the C terminal	Addgene (#155297), from Raffaele De Francesco's lab
3.	psPAX2	Lentiviral packaging plasmid	Packaging vector	Addgene (#12260) from

				Didier Trono's lab
4.	pMD2.G	Expression of VSV-G glycoprotein	Envelope plasmid	Addgene (#12259) from Didier Trono's lab
5.	pBJ5-VSV-G	Expression of HA-tagged VSV-G glycoprotein	Envelope plasmid	Gifted by Prof. Massimo Pizzato
6.	pcDNA3.1BS(-)	Control for transfection and vector backbone for cloning	Mammalian expression vector	This study
7.	pWPI-IRES-Puro-Ak-ACE2-TMPRSS2	Source for human ACE2	Puromycin resistance marker	Addgene (#154987) from Sonja Best's lab
8.	ACE2 in pScalps Hygro	Establishment of ACE2+ cell line		This study
9.	ACE2-IgFc	Expression of ACE2-IgFc construct	6XHistidine tag at the C-terminus	This study
10.	Nsp1 in pcDNA3.1BS(-)	Expression of SARS-CoV-2's non-structural protein 1	2X Strep Tag	This study
11.	Nsp2 in pcDNA3.1BS(-)	Expression of SARS-CoV-2's non-structural protein 2	2X Strep Tag	This study
12.	Nsp4 in pcDNA3.1BS(-)	Expression of SARS-CoV-2's non-structural protein 4	2X Strep Tag	This study
13.	Nsp5 in pcDNA3.1BS(-)	Expression of SARS-CoV-2's non-structural protein 5	2X Strep Tag	This study
14.	Nsp7 in pcDNA3.1BS(-)	Expression of SARS-CoV-2's non-structural protein 7	2X Strep Tag	This study
15.	Nsp8 in pcDNA3.1BS(-)	Expression of SARS-CoV-2's non-structural protein 8	2X Strep Tag	This study
16.	Nsp10 in	Expression of SARS-	2X Strep Tag	This study

	pcDNA3.1BS(-)	CoV-2's non-structural protein 10		
17.	Nsp11 in pcDNA3.1BS(-)	Expression of SARS-CoV-2's non-structural protein 1	2X Strep Tag	This study
18.	Nsp12 in pcDNA3.1BS(-)	Expression of SARS-CoV-2's non-structural protein 12	2X Strep Tag	This study
19.	Nsp13 in pcDNA3.1BS(-)	Expression of SARS-CoV-2's non-structural protein 13	2X Strep Tag	This study
20.	Nsp14 in pcDNA3.1BS(-)	Expression of SARS-CoV-2's non-structural protein 14	2X Strep Tag	This study
21.	Nsp15 in pcDNA3.1BS(-)	Expression of SARS-CoV-2's non-structural protein 15	2X Strep Tag	This study
22.	ORF3a in pcDNA3.1BS(-)	Expression of SARS-CoV-2's open reading frame 3a	2X Strep Tag	This study
23.	ORF3b in pcDNA3.1BS(-)	Expression of SARS-CoV-2's open reading frame 3b	2X Strep Tag	This study
24.	ORF6 in pcDNA3.1BS(-)	Expression of SARS-CoV-2's open reading frame 6	2X Strep Tag	This study
25.	ORF7a in pcDNA3.1BS(-)	Expression of SARS-CoV-2's open reading frame 7a	2X Strep Tag	This study
26.	ORF7b in pcDNA3.1BS(-)	Expression of SARS-CoV-2's open reading frame 7b	2X Strep Tag	This study
27.	ORF8 in pcDNA3.1BS(-)	Expression of SARS-CoV-2's open reading frame 8	2X Strep Tag	This study
28.	ORF9b in pcDNA3.1BS(-)	Expression of SARS-CoV-2's open reading frame 9b	2X Strep Tag	This study

29.	ORF9c in pcDNA3.1BS(-)	Expression of SARS-CoV-2's open reading frame 9c	2X Strep Tag	This study
30.	ORF10 in pcDNA3.1BS(-)	Expression of SARS-CoV-2's open reading frame 10	2X Strep Tag	This study
31.	M protein in pcDNA3.1BS(-)	Expression of SARS-CoV-2's membrane protein	2X Strep Tag	This study
32.	E protein in pcDNA3.1BS(-)	Expression of SARS-CoV-2's envelope protein	2X Strep Tag	This study
33.	N protein in pcDNA3.1BS(-)	Expression of SARS-CoV-2's nucleocapsid protein	2X Strep Tag	This study
34.	pScalps Hygro	Lentiviral vector backbone for selection of transduced cells by hygromycin	Hygromycin resistance marker and an MCS for expression under SFFV promoter	This study
35.	pTZ57R	Scaffold for cloning of ACE2-IgFc	Cloning vector	Fermentas/Thermo Scientific
36.	pcDNA3.1-Spike G614	The spike D614G variant	Envelope plasmid	This study
37.	NCA Zsgreen	For MLV core studies ~~packaging plasmid~~	Spike-pseudotyping using MLV system	Gifted by Prof. Massimo Pizzato

Table S2: Sequences of ACE2-IgFc:

Nucleotide sequence of ACE2-IgFc:	5'-<br>ATGTCAAGCTCTTCCTGGCTCCTTCTCAGCCTTGTTGCTGTAACTGCTGC<br>TCAGTCCACCATTGAGGAACAGGCCAAGACATTTTTGGACAAGTTTAACC<br>ACGAAGCCGAAGACCTGTTCTATCAAAGTTCACTTGCTTCTTGGAATTATA<br>ACACCAATATTACTGAAGAGAATGTCCAAAACATGAATAATGCTGGGGAC<br>AAATGGTCTGCCTTTTTAAAGGAACAGTCCACACTTGCCCAAATGTATCC<br>ACTACAAGAAATTCAGAATCTCACAGTCAAGCTTCAGCTGCAGGCTCTTC

	AGCAAAATGGGTCTTCAGTGCTCTCAGAAGACAAGAGCAAACGGTTGAA<br>CACAATTCTAAATACAATGAGCACCATCTACAGTACTGGAAAAGTTTGTAA<br>CCCAGATAATCCACAAGAATGCTTATTACTTGAACCAGGTTTGAATGAAAT<br>AATGGCAAACAGTTTAGACTACAATGAGAGGCTCTGGGCTTGGGAAAGC<br>TGGAGATCTGAGGTCGGCAAGCAGCTGAGGCCATTATATGAAGAGTATG<br>TGGTCTTGAAAAATGAGATGGCAAGAGCAAATCATTATGAGGACTATGGG<br>GATTATTGGAGAGGAGACTATGAAGTAAATGGGGTAGATGGCTATGACTA<br>CAGCCGCGGCCAGTTGATTGAAGATGTGGAACATACCTTTGAAGAGATTA<br>AACCATTATATGAACATCTTCATGCCTATGTGAGGGCAAAGTTGATGAAT<br>GCCTATCCTTCCTATATCAGTCCAATTGGATGCCTCCCTGCTCATTTGCTT<br>GGTGATATGTGGGGTAGATTTTGGACAAATCTGTACTCTTTGACAGTTCC<br>CTTTGGACAGAAACCAAACATAGATGTTACTGATGCAATGGTGGACCAGG<br>CCTGGGATGCACAGAGAATATTCAAGGAGGCCGAGAAGTTCTTTGTATCT<br>GTTGGTCTTCCTAATATGACTCAAGGATTCTGGGAAAATTCCATGCTAAC<br>GGACCCAGGAAATGTTCAGAAAGCAGTCTGCCATCCCACAGCTTGGGAC<br>CTGGGGAAGGGCGACTTCAGGATCCTTATGTGCACAAAGGTGACAATGG<br>ACGACTTCCTGACAGCTCATCATGAGATGGGGCATATCCAGTATGATATG<br>GCATATGCTGCACAACCTTTTCTGCTAAGAAATGGAGCTAATGAAGGATT<br>CCATGAAGCTGTTGGGGAAATCATGTCACTTTCTGCAGCCACACCTAAGC<br>ATTTAAAATCCATTGGTCTTCTGTCACCCGATTTTCAAGAAGACAATGAAA<br>CAGAAATAAACTTCCTGCTCAAACAAGCACTCACGATTGTTGGGACTCTG<br>CCATTTACTTACATGTTAGAGAAGTGGAGGTGGATGGTCTTTAAAGGGGA<br>AATTCCCAAAGACCAGTGGATGAAAAAGTGGTGGGAGATGAAGCGAGAG<br>ATAGTTGGGGTGGTGGAACCTGTGCCCCATGATGAAACATACTGTGACC<br>CCGCATCTCTGTTCCATGTTTCTAATGATTACTCATTCATTCGATATTACA<br>CAAGGACCCTTTACCAATTCCAGTTTCAAGAAGCACTTTGTCAAGCAGCT<br>AAACATGAAGGCCCTCTGCACAAATGTGACATCTCAAACTCTACAGAAGC<br>TGGACAGAAACTGTTCAATATGCTGAGGCTTGGAAAATCAGAACCCTGGA<br>CCCTAGCATTGGAAAATGTTGTAGGAGCAAAGAACATGAATGTAAGGCCA<br>CTGCTCAACTACTTTGAGCCCTTATTTACCTGGCTGAAAGACCAGAACAA<br>GAATTCTTTTGTGGGATGGAGTACCGACTGGAGTCCATATGCAGACCAAA<br>GCATCAAAGTGAGGATAAGCCTAAAATCAGCTCTTGGAGATAAAGCATAT<br>GAATGGAACGACAATGAAATGTACCTGTTCCGATCATCTGTTGCATATGC<br>TATGAGGCAGTACTTTTTAAAAGTAAAAAATCAGATGATTCTTTTTGGGGA<br>GGAGGATGTGCGAGTGGCTAATTTGAAACCAAGAATCTCCTTTAATTTCT<br>TTGTCACTGCACCTAAAAATGTGTCTGATATCATTCCTAGAACTGAAGTTG<br>AAAAGGCCATCAGGATGTCCCGGAGCCGTATCAATGATGCTTTCCGTCT<br>GAATGACAACAGCCTAGAGTTTCTGGGGATACAGCCAACACTTGGACCT<br>CCTAACCAGCCCCCTGTTTCCCTCGAGGACAAAACTCACAAATGCCCAC<br>CGTGCCCAGCACCTGAACTCCtGGGGGGACCGTCAGTCTTCCTCTTCCC<br>CCCAAAACCCAAggAtaCCCTTATGATTTCCCGGACCCCTGAGGTCACGTG<br>CGTGGTGGTGGACGTGAGCCACGAAGACCCCGAGGTCCAGTTCAAGTG<br>GTACGTGGACGGCGTGGAGGTGCATAATGCCAAGACAAAGCTGCGGGA<br>GGAGCAGTACAACAGCACGTTCCGTGTGGTCAGCGTCCTCACCGTCCTG<br>CACCAGGACTGGCTGAACGGCAAGGAGTACAAGTGCAAGGTCTCCAACA<br>AAGCCCTCCCAGCCCCCATCGAGAAAACCATCTCCAAAGCCAAAGGGCA<br>GCCCCGAGAACCACAGGTGTACACCCTGCCCCCATCCCGGATGAGCT

	GACCAAGAACCAGGTCAGCCTGACCTGCCTGGTCAAAGGCTTCTATCCC AGCGACATCGCCGTGGAGTGGGAGAGCAATGGGCAGCCGGAGAACAAC TACAAGACCACGCCTCCCGTGCTGGACTCCGACGGCTCCTTCTTCCTCT ACAGCAAGCTCACCGTGGACAAGAGCAGGTGGCAGCAGGGGAACGTCT TCTCATGCTCCGTGATGCATGAGGGTCTGCACAACCACTACACGCAGAA GAGCCTCTCCCTGTCTCCGGGTAAAGGCGGCCATCATCACCATCACCAT TAA-3’
Translation of ACE2-IgFc:	MSSSSWLLLSLVAVTAAQSTIEEQAKTFLDKFNHEAEDLFYQSSLASWNYNT NITEENVQNMNNAGDKWSAFLKEQSTLAQMYPLQEIQNLTVKLQLQALQQN GSSVLSEDKSKRLNTILNTMSTIYSTGKVCNPDNPQECLLLEPGLNEIMANSL DYNERLWAWESWRSEVGKQLRPLYEEYVVLKNEMARANHYEDYGDYWRG DYEVNGVDGYDYSRGQLIEDVEHTFEEIKPLYEHLHAYVRAKLMNAYPSYIS PIGCLPAHLLGDMWGRFWTNLYSLTVPFGQKPNIDVTDAMVDQAWDAQRIF KEAEKFFVSVGLPNMTQGFWENSMLTDPGNVQKAVCHPTAWDLGKGDFRI LMCTKVTMDDFLTAHHEMGHIQYDMAYAAQPFLLRNGANEGFHEAVGEIMS LSAATPKHLKSIGLLSPDFQEDNETEINFLLKQALTIVGTLPFTYMLEKWRWM VFKGEIPKDQWMKKWWEMKREIVGVVEPVPHDETYCDPASLFHVSNDYSFI RYYTRTLYQFQFQEALCQAAKHEGPLHKCDISNSTEAGQKLFNMLRLGKSE PWTLALENVVGAKNMNVRPLLNYFEPLFTWLKDQNKNSFVGWSTDWSPYA DQSIKVRISLKSALGDKAYEWNDNEMYLFRSSVAYAMRQYFLKVKNQMILFG EEDVRVANLKPRISFNFFVTAPKNVSDIIPRTEVEKAIRMSRSRINDAFRLNDN SLEFLGIQPTLGPPNQPPVSLEDKTHKCPPCPAPELLGGPSVFLFPPKPKDTL MISRTPEVTCVVVDVSHEDPEVQFKWYVDGVEVHNAKTKLREEQYNSTFRV VSVLTVLHQDWLNGKEYKCKVSNKALPAPIEKTISKAKGQPREPQVYTLPPS RDELTKNQVSLTCLVKGFYPSDIAVEWESNGQPENNYKTTPPVLDSDGSFFL YSKLTVDKSRWQQGNVFSCSVMHEGLHNHYTQKSLSLSPGKGGHHHHHH
Legend:	N-terminal human ACE2 sequence; Modified C-terminal soluble ACE2 Fragment; Cloning sites; IgG Fc Fragment; 6XHistidine tag

Supplementary figure-1

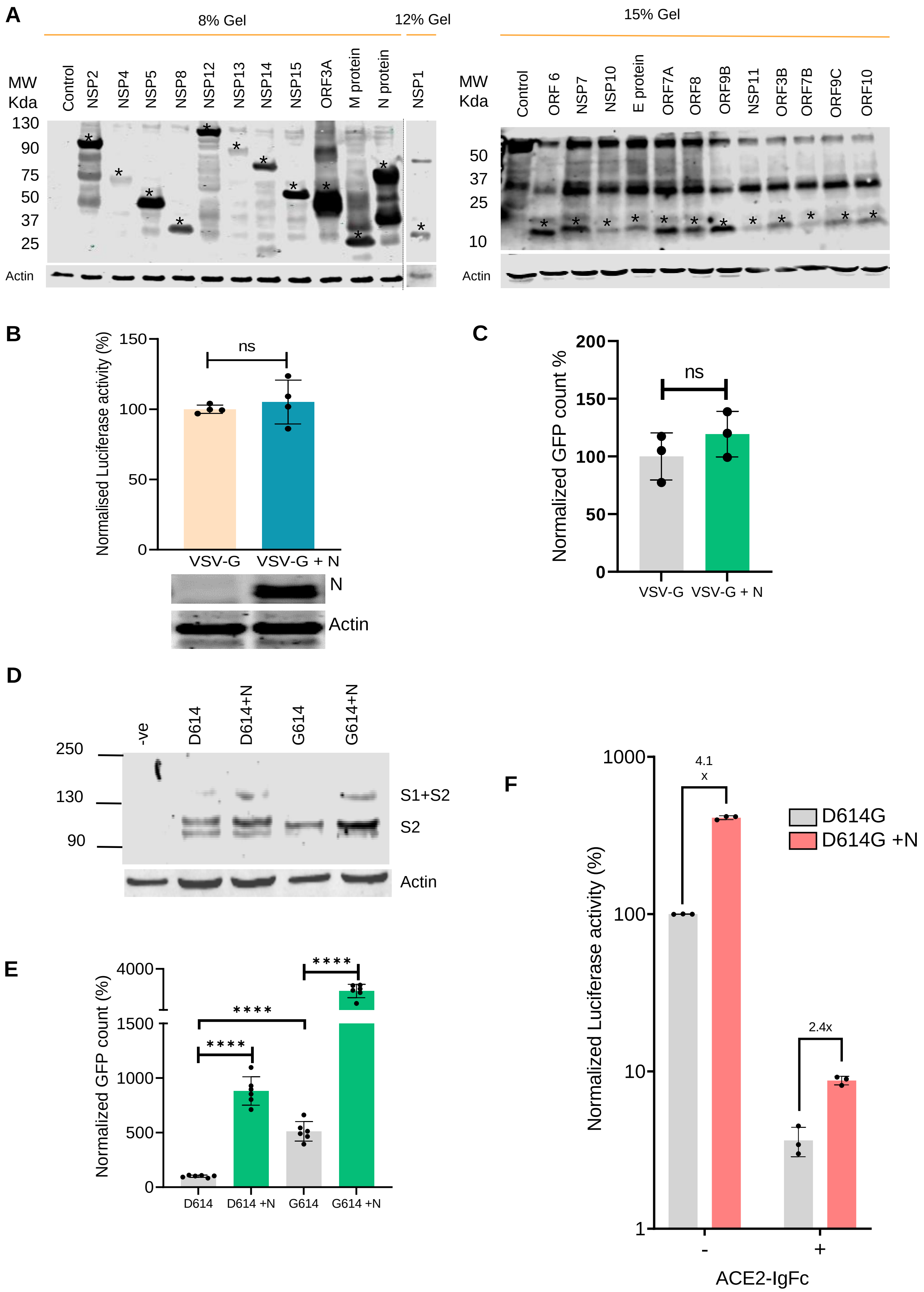

Supplement: Supplementary file 1 [file DataSheet_1.pdf]
